# Erratum: RENATA study—Latin American prospective experience: clinical outcome of patients treated with palbociclib in hormone receptor-positive metastatic breast cancer—real-world use

**DOI:** 10.3332/ecancer.2020.1087

**Published:** 2020-08-07

**Authors:** Fernando Petracci, Gonzalo Gomez Abuin, Alejandra Pini, Matías Chacón

**Affiliations:** 1 Breast Cancer Department, Instituto Alexander Fleming and Sanatorio Las Lomas, Buenos Aires 1428, Argentina; 2 Oncology Service, Hospital Alemán, Buenos Aires 1428, Argentina; 3 Oncology Service, Hospital Militar Central and Sanatorio Las Lomas, Buenos Aires 1428, Argentina; 4 Oncology Service Chair, Instituto Alexander Fleming, Buenos Aires 1642, Argentina

On page 3, the number 72.5 in the following sentence replaces the number 37.5 which was incorrect:

'The Overall CB was 82.5%, higher in first line than in second line, 85.9% and 62.5%, respectively. The ORR in first line were: 4.7% CR, 40.6% PR and 40.6% SD. In second line the ORR were: 25% PR, 37.5% SD and no CR.'

## Conflicts of interest

There are no conflicts of interest.

## Funding statement

This research did not receive any specific grant from funding agencies in the public, commercial or not-for-profit sectors.

